# T cell epitopes of SARS-CoV-2 spike protein and conserved surface protein of *Plasmodium malariae* share sequence homology

**DOI:** 10.1515/biol-2021-0062

**Published:** 2021-06-23

**Authors:** Md. Mehedi Hassan, Shirina Sharmin, Jinny Hong, Hoi-Seon Lee, Hyeon-Jin Kim, Seong-Tshool Hong

**Affiliations:** Department of Biomedical Sciences and Institute for Medical Science, Jeonbuk National University Medical School, Jeonju, Jeonbuk 54907, South Korea; JINIS BDRD Institute, JINIS Biopharmaceuticals Inc., 224 Wanjusandan 6-Ro, Bongdong, Wanju, Jeonbuk 55315, South Korea; SNJ Pharma Inc., 1124 West Carson St. MRL Bldg 3F, BioLabs LA in The Lundquist Institute for Biomedical Innovation at Harbor-UCLA Medical Center, Torrance, CA 90502, United States of America; Department of Bioenvironmental Chemistry, Jeonbuk National University, Jeonju, Jeonbuk 54896, South Korea

**Keywords:** SARS-CoV-2, spike glycoprotein, prototype, proteome, phylogenetic analysis, malaria

## Abstract

Since its emergence in late 2019, severe acute respiratory syndrome coronavirus 2 (SARS-CoV-2) has been spreading remarkably fast worldwide. Effective countermeasures require the rapid development of data and tools to monitor its spread and better understand immunogenic profile. However, limited information is available about the tools and target of the immune responses to SARS-CoV-2. In this study, we excogitated a new approach for analyzing phylogenetic relationships by using the whole prototype proteome sequences. Phylogenetic analysis on the whole prototype proteome sequences showed that SARS-CoV-2 was a direct descendant of Bat-CoV and was closely related to Pangolin-CoV, Bat-SL-CoV, and SARS-CoV. The pairwise comparison of SARS-CoV-2 with Bat-CoV showed an unusual replacement of the motif consisting of seven amino acids (NNLDSKV) within the spike protein of SARS-CoV-2. The replaced motif in the spike protein of SARS-CoV-2 was found in 12 other species, including a conserved surface protein of a malaria-causing pathogen, *Plasmodium malariae*. We further identified the T and B cell epitope sequence homology of SARS-CoV-2 spike protein with conserved surface protein of *P. malariae* using the Immune Epitope Database and Analysis Resource (IEDB). The shared immunodominant epitopes may provide immunity against SARS-CoV-2 infection to those previously infected with *P. malariae*.

## 1 Introduction

Coronaviruses (CoVs) are the predominant cause of the common cold widely present in nature with a broad spectrum of hosts. Although the viruses frequently infect humans, the natural host of CoVs are animals, and thereby all of the CoVs for the common colds, HCoV-229E, HCoV-NL63, HCoV-OC43, and HCoV-HKU1, have zoonotic origins [1]. The genomes of CoVs consist of a single-stranded, positive RNA of 26,000–32,000 base pairs and a variable number (from 6 to 11) of open reading frames [2]. Because of their considerable size with the characteristics of the RNA genomes, CoVs can frequently mutate to escape their natural hosts, causing severe diseases in humans. Outbreaks of SARS-CoV in 2003 and MERS-CoV in 2012 are well-known examples. Currently, another severe pathogenic novel coronavirus, SARS-CoV-2, has emerged and caused a global pandemic [3,4].

CoVs are RNA viruses with a positive-sense single-stranded genome, and their RNA genomes are vulnerable to natural mutations like other RNA viruses, which cause significant genetic diversity. A high degree of genetic diversity in CoVs makes it challenging to find the phylogenetic relationship of CoVs. Understanding the phylogenetic relationship of SARS-CoV-2 with other CoVs is essential for identifying its host to prevent the next outbreak. Because of the ambiguous results by genome comparison approaches, an alignment-free method called natural vector was adopted to investigate the phylogeny of SARS-CoV-2 [5]. However, the frequency of the original sequence always predominates the mutated sequence if the genomes of individuals were compared within the same species. Therefore, it is possible to construct a prototype proteome of a species by identifying a prevalent amino acid (aa) at each position of proteome after multiple alignments of individual proteomes for the analysis of the phylogenetic relationships.

CoVs contain four different structural proteins, including spike (S), envelop (E), membrane (M), and nucleocapsid (N) proteins, and S protein plays the most critical roles in viral attachment and entry [6]. The S protein first binds to the host receptor through the receptor-binding domain (RBD) in the S1 subunit and then makes entry by fusing the viral and host membranes through the S2 subunit [7,8]. Thus, the S protein of SARS-CoV-2 becomes an attractive target for the development of virus attachment inhibitors, neutralizing antibodies, and vaccines [9,10,11]. However, there is limited information available on which parts of the SARS-CoV-2 are recognized by human immune responses. Thus, the knowledge of the potential immunogenic profile of SARS-CoV-2 is of immediate relevance and would assist in vaccine design and facilitate the evaluation of vaccine candidate immunogenicity, monitoring of mutational events, and the epitope escape during transmission.

In this study, we constructed a prototype proteome sequence of SARS-CoV-2 and its related CoVs species to understand the biological characteristics and phylogenetic relationship. The phylogeny of SARS-CoV-2 and Bat-CoV prompted us to explore the distribution of unique NNLDSKV peptides on different species, including the conserved surface protein of *P. malariae*. Here, we used IEDB resources to find out the T and B cell epitopes of the conserved surface protein of *P. malariae,* which has NNLDSKV sequence homology with SARS-CoV-2 spike glycoprotein. Furthermore, we analyzed the possible shared immunogenic regions of the conserved surface protein of *P*. *malariae* and SARS-CoV-2 spike glycoprotein using immunoinformatic approaches.

## 2 Methods

### 2.1 Genome datasets

The SARS-CoV-2, Bat-CoV, Pan-CoV, Bat-SL-CoV, and SARS-CoV genome sequences were obtained from GenBank using Blastn and the GISAID (https://www.gisaid.org) databases, with data kindly deposited by the submitters (Supplementary Table S1).

### 2.2 Prediction of protein-coding genes in genome sequences

The protein-encoding genes of the genome sequences of each viral species were predicted by the online servers of GeneMarkS (http://exon.gatech.edu/GeneMark/genemarks.cgi) and ORFfinder (https://www.ncbi.nlm.nih.gov/orffinder/) with a manual check.

### 2.3 *In silico* translation of protein-coding genes

The protein-coding genes of the genome sequences of each viral species were translated using the ExPASy protein translation tool (https://web.expasy.org/translate/).

### 2.4 Determination of prototype proteome sequences

To generate prototype sequences, the different proteome sequences of each viral species obtained from their genome sequences (Supplementary Table S1) were aligned using the FFT-NS-2 algorithm in MAFFT (version v7) [12]. Each amino acid position was investigated with the manual check from the corresponding multiple sequence alignment dataset, and the most prevalent amino acids were chosen for the prototype sequence. The prototype aa sequences of open reading frame ORF1a, ORF1ab, spike (S), 3a, 3b, envelope (E), membrane (M), 6, 7a, 7b, 8, Nucleocapsid (N), 9b, and 14 for all coronavirus type were determined by the following method.

Let S = (*s*_1_, *s*_2_, …, *s*_*i*_, …, *s*_*n*_) be an aa sequence of length *n*, where *s*_*i*_ ∈ {0, *A*, *C*, *D*, *E*, *F*, *G*, *H*, *I*, *K*, *L*, *M*, *N*, *P*, *Q*, *R*, *S*, *T*, *V*, *W*, *Y*}. Let *s[k][i]* be the location of the *i*th occurrence of aa *k*. PS = (*ps*_1_, *ps*_2_, …, *ps*_*i*_, …, *ps*_*n*_) is a prototype aa sequence with a specific aa *ps* at the *i*th occurrence was determined by selecting *k* with the largest *n*. *nk*_*i*_ =]∑*k*_*i*,_ where *k* is a specific aa sequence of the individual protein sequences at the *i*th occurrence.

### 2.5 Sequence similarity search of NNLDSKV motif

The spike protein of SARS-CoV-2 was overlappingly defragmented in 9 aa sequence unit. The defragmented aa sequences were used to identify the matched sequences in GenBank using Protein Blastp tools (https://blast.ncbi.nlm.nih.gov/Blast.cgi).

### 2.6 Phylogenetic analysis, 3D homology modeling, and annotation

All ORF of SARS-CoV-2 were aligned against Bat-CoV, Pan-CoV, Bat-SL-CoV, and SARS-CoV using the FFT-NS-2 algorithm in MAFFT (version v7) [12]. Maximum likelihood phylogenies were estimated using Unipro UGENE bioinformatics toolkits [13]. To generate the 3D model, the query amino acid sequences were run in the SWISS-MODEL protein homology-modeling server to produce several best-fit homo-trimeric or monomeric protein models based on multiple template alignment [14]. The homotrimer 3D model of Bat-CoV and SARS-CoV-2 spike protein with the highest sequence identity coverage was used in this study. Furthermore, the 3D model of the NNLDSKV motif in the SARS-CoV-2 spike and *P. malariae* was built by the SWISS-MODEL protein homology-modeling server. The PDB sequence file of the NNLDSKV motif 3D model was then processed in UCSF CHIMERA to generate the surface structure [15].

### 2.7 Prediction of immunodominant epitopes of SARS-CoV-2

The T and B cell epitopes of SARS-CoV-2 from immunodominant regions were determined based on sequence-shared identities with the closely related SARS-CoV using parallel bioinformatics approaches by Grifoni et al. [16]. In this study, epitope sequences of SARS-CoV-2 spike glycoprotein were retrieved and matched with our prototype SARS-CoV-2 spike glycoprotein sequence and used to identify common immunodominant epitopes of *P. malariae* conserved surface protein (XP_028861348.1).

### 2.8 Prediction of T and B cell epitopes of the conserved surface protein of *P. malariae* and homology analysis with SARS-CoV-2 spike glycoprotein

The T and B cell epitopes for *P*. *malariae* conserved surface protein were determined by searching the Immune Epitope Database and Analysis Recourse (IEDB, http://www.iedb.org/) in the middle of April 2021. The prediction of B cell epitopes of *P*. *malariae* conserved surface protein was carried out using Bepipred linear epitope prediction algorithm by setting the threshold at 0.55 embedded in the B cell prediction analysis tools available in IEDB [17]. The CD4 T cell epitopes of *P*. *malariae* conserved surface protein were analyzed using the combined method by setting the threshold at 90 embedded in T cell epitope prediction tools available in IEDB [18]. The predictions of peptide binding of *P*. *malariae* conserved surface protein immunodominant T cell epitopes to MHC class I molecules were calculated by MHC-I binding predictions tool using IEDB recommended 2020.09 (NetMHCpan EL 4.1) method with selected HLA allele reference set, and outputs with lowest percentile rank were chosen from the default results (low percentile rank = good binders) [19]. The maps of the T cell epitopes identified from *P*. *malariae* conserved surface protein were analyzed based on selected sequence identity to the given parent antigen (*P. malariae* conserved surface protein) and nonparental sequence (SARS-CoV-2 spike glycoprotein) using ImmunomeBrowser tool available in IEDB [20]. The sequence seminaries within the T cell epitopes of SARS-CoV-2 and *P. malariae* conserved surface protein were analyzed using the Epitope cluster analysis tool using the threshold of 70 available in IEDB analysis resource [21]. The degree of conservancy between T cell epitopes (peptide core sequence) of *P*. *malariae* conserved surface protein and T cell epitopes of SARS-CoV-2 were calculated by the epitope conservancy analysis tool, and data were represented as identity of percentage (%) [22]. Four or five amino acid shared residues were considered significant [23].

## 3 Results

### 3.1 Comparison of the whole prototype proteomes of coronaviruses to SARS-CoV-2 revealed the phylogeny without showing ambiguity

Identifying an animal vector transmitting an infectious disease to humans could be significant as much as understanding at the person-to-person transmission stage to control the disease, meaning that it is vital to identify the correct host. To overcome the current ambiguity of various genome analysis approaches, we excogitated a completely new approach comparing whole prototype proteomes of species to investigate the phylogeny of SARS-CoV-2. To construct the whole prototype proteomes of viral species related to SARS-CoV-2, we first collected all the individual genome sequences of SARS-CoV-2 as well as its related viral species by the BLAST sequence similarity search from all of the publicly available genome databases (Supplementary Table S1). The genome sequences of the individual viruses were converted into protein sequences. After *in silico* translation of the individual viruses, the proteomes of individual viruses of the same viral species were aligned through multiple sequence alignments (MSAs) followed by identifying the most prevalent aa in each position within the same species to determine the prototype proteome sequences for each viral species.

**Figure 1:**
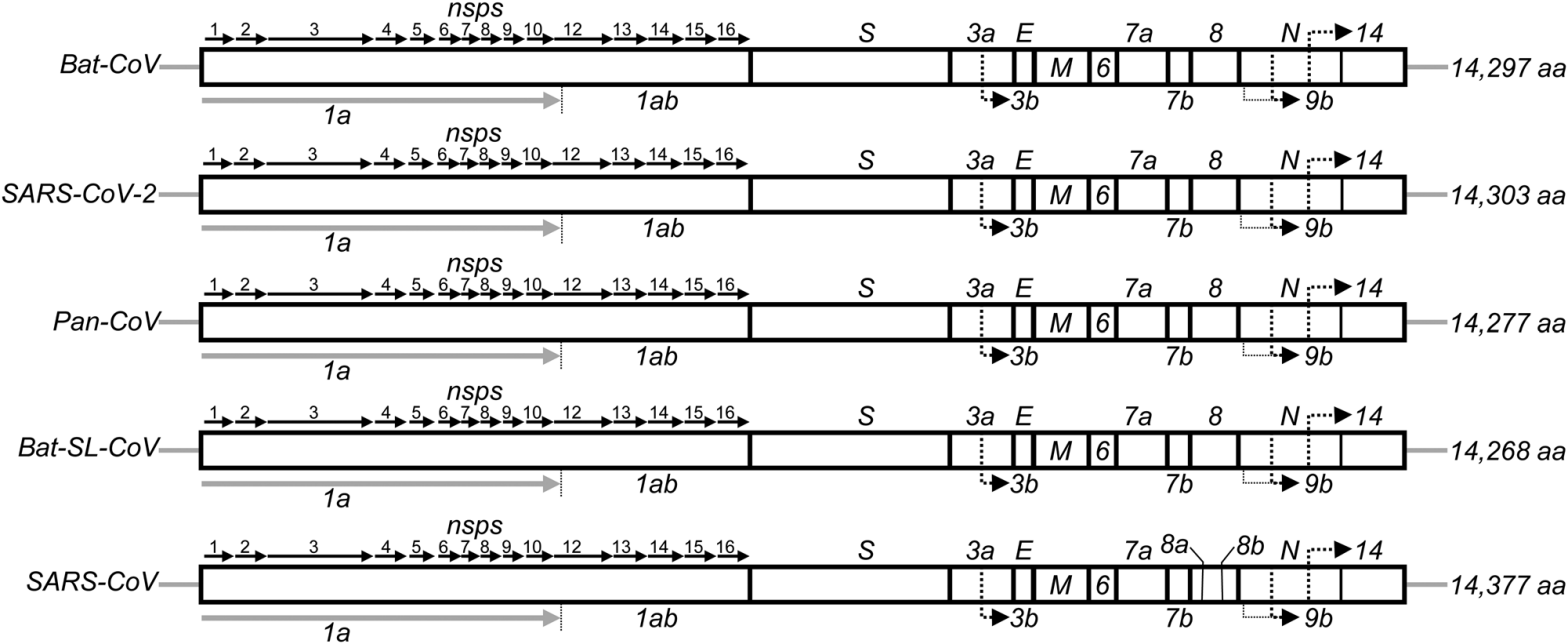
Sequence alignment and genome organization of Bat-CoV, SARS-CoV-2, Pan-CoV, Bat-SL-CoV, and SARS-CoVs. The gene ORF1ab encodes the pp1ab protein that contains 15 predicted nonstructural proteins (nsps). The structural proteins are encoded by Spike (S), Envelope (E), and Nucleocapsid (N) genes. The protein-encoding genes of CoVs genome were predicted by GeneMarks and ORFfinder online server with a manual check.

The prototype aa sequences of the proteomes of SARS-CoV-2 and its related species, Bat-CoV, Pan-CoV, Bat-SL-CoV, and SARS-CoV, are represented in Supplementary Figure S1 and Supplementary Table S2. As shown in Figure 1, the prototype proteome of SARS-CoV-2 has consisted of 14 proteins, similar to other beta-coronaviruses. The 5′-terminal two-thirds of the genome encodes replicase polyprotein 1ab (pp1ab) with a length of 7,096 aa and contains 15 predicted nonstructural proteins (Supplementary Table S3). The 3′ terminus encodes four structural proteins and other nonstructural proteins, including spike glycoprotein (S), ORF3a, ORF3b, envelope small membrane protein (E), membrane protein (M), ORF6, ORF7a, ORF7b, ORF8, ORF9b, nucleocapsid protein (N), and ORF14 in order.

**Figure 2:**
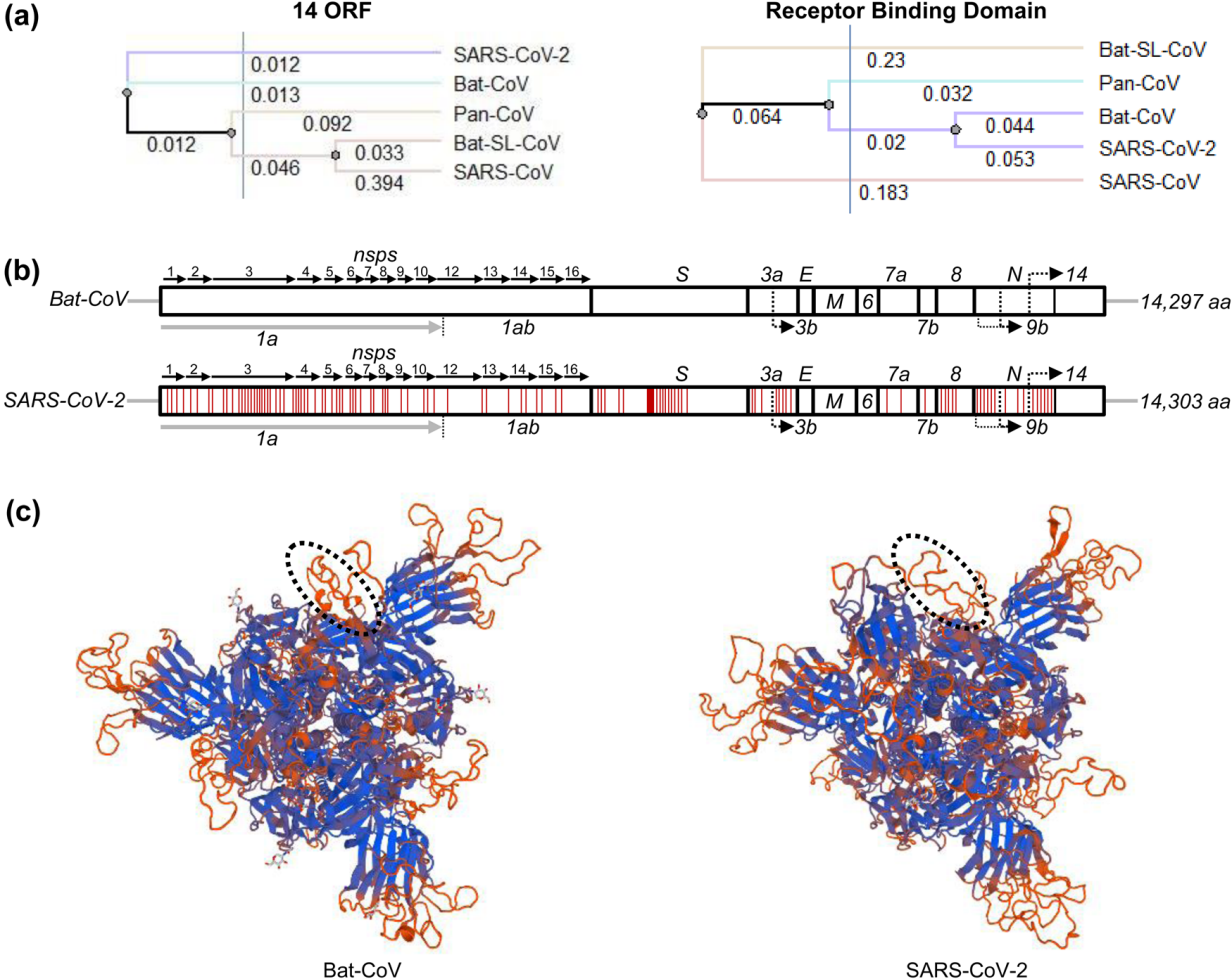
Comparison of SARS-CoV-2 and Bat-CoVs. (a) The phylogeny of SARS-CoV-2 based on the 14 ORF and receptor-binding domain (RBD) sequences of Bat-CoV, Pan-CoV, Bat-SL-CoV, and SARS-CoV. Phylogenies were estimated by the neighbor-joining method using Unipro UGENE bioinformatics toolkits. (b) Organization of genes in SARS-CoV-2 and Bat-CoVs. The distribution of mutated amino acids in SARS-CoV-2 compare to Bat-CoV are represented with red lines. (c) Homotrimer 3D model of Bat-CoV and SARS-CoV-2 spike protein constructed using the SWISS-MODEL protein homology-modeling server. Circle showing the altered surface structure in the spike region of SARS-CoV-2 for antigen binding.

The phylogenetic relationship of the total aa sequences of the whole prototype proteomes of the five viral species was analyzed by the neighbor-joining method using Unipro UGENE bioinformatics toolkits [13]. As shown in Figure 2a, SARS-CoV-2 is a direct descendant of Bat-CoV and did not originate from Pan-CoV, Bat-SL-CoV, and SARS-CoV. That suggests that pangolin may act as an intermediate host [24]. The sequence alignment of the prototype amino acid sequences by using MSA with MAFFT program [12] showed that the aa sequence of SARS-CoV-2 shared 98.67% sequence similarity with Bat-CoV, while 94.51, 94.35, and 82.93% of the sequences of SARS-CoV-2 were identical with that of Pan-CoV, Bat-SL-CoV, and SARS-CoV, respectively (Supplementary Table S4). The aa sequence similarities were well matched with their phylogenetic distances.

### 3.2 Pairwise analysis of SARS-CoV-2 and Bat-CoV prototype proteome sequences identified a unique peptide (NNLDSKV) in SARS-CoV-2 spike glycoprotein

To investigate the sequence dissimilarities between SARS-CoV-2 and Bat-CoV, we performed pairwise sequence analysis between the prototype aa sequences of SARS-CoV-2 and Bat-CoV. The pairwise aa sequence analysis showed that both virus genomes encode 14 genes (Figure 2b). The aa sequence mismatches were randomly distributed except for the consecutive 7-aa alteration (^439^NNLDSKV^445^) in the spike protein. Since genetic mutation is a random process, the mutated points are expected to be distributed randomly throughout the genome. Considering the general nature of mutations, the consecutive 7-aa sequence difference in the spike protein was peculiar enough for us to speculate that an unusual event happened during the emergence of SARS-CoV-2 from Bat-CoV. 3D models of the spike proteins were made to analyze the consequence of the alteration to the SARS-CoV-2 spike protein structure. The spike protein of CoV is the surface protein that binds to a receptor on the host cell surface. The spike protein consists of three large domains: a large ectodomain, a single-pass transmembrane anchor, and a short intracellular tail [25]. The ectodomain is further divided into a receptor-binding subunit S1 and a membrane-fusion subunit S2. Coronavirus first binds to a receptor on the host cell surface through its S1 subunit and then fuses the viral and the host membranes through its S2 subunit, meaning that the S1 domain plays the most critical role in the virus’s invasion into its host [26,27]. As shown in Figure 2c, altering the consecutive 7-aa on the outer layer of the spike protein’s S1 domain did not affect the overall structural integrity of the spike protein. The 3D modeling also showed that the consecutive 7-aa alteration resulted in a new motif occupying more space in the S1 domain than its original structure by transforming a partial alpha-helical structure to a random coil. Since the S1 domain acts as the binding domain for the entry of the virus, enlargement of its space and adaptation of different conformation in the outermost layer surface of the S1 domain would endow SARS-CoV-2 to expand its host range.

**Figure 3:**
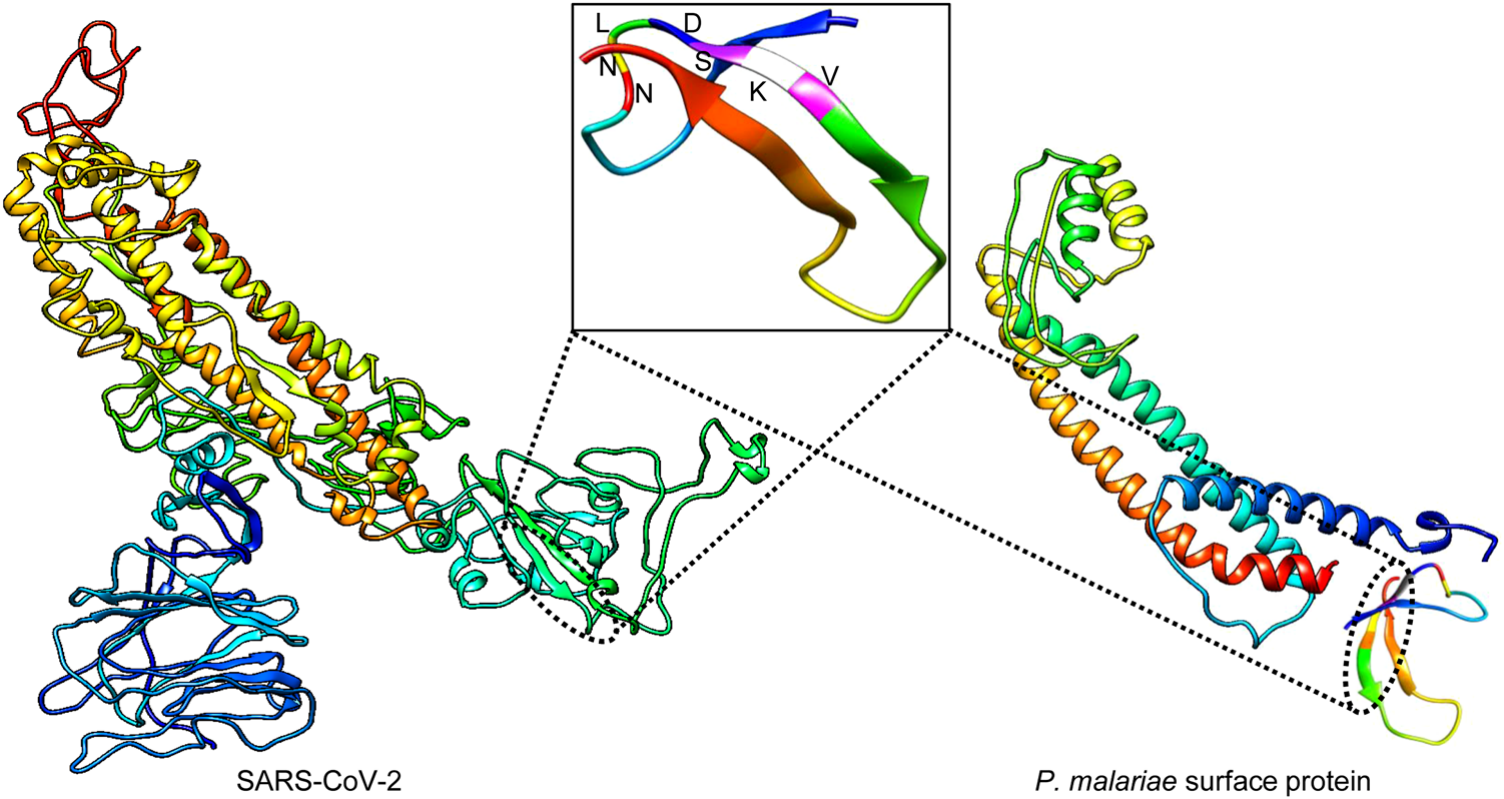
The SARS-CoV-2 spike glycoprotein showed NNLDSKV motif identity with conserved surface protein of *P*. *malariae*. The 3D model for the NNLDSKV motif of SARS-CoV-2 and *P. malariae* surface protein was built by the SWISS-MODEL protein homology-modeling server, and the PDB sequence was analyzed by UCSF CHIMERA software. The orientation of 7-amino acids in the NNLDSKV motif is shown in the box with different colors.

The unusual genetic alteration in the spike protein between SARS-CoV-2 and Bat-CoV motivated us to investigate the unique nature of the 7-aa motif (^439^NNLDSKV^445^). To trace down its distribution, we overlappingly defragmented the aa sequence of the spike protein of SARS-CoV-2 into 7 aa units and performed a peptide sequence search in the publicly available protein databases. All of the aa sequences matched with the spike proteins of coronaviruses except for the 7-aa motif. Interestingly, the 7-aa motif (^439^NNLDSKV^445^) of SARS-CoV-2 was found in 12 other species, including *P. malariae,* which causes malaria to humans (Table 1). As presented in Table 1, the 7-aa motif is primarily present in the surface proteins of simple organisms. The motif’s widespread in simple organisms as a surface protein suggests that the motif plays a significant role on the surface of the organisms. It is also worth noting that the NNLDSKV motif was located at aa 449–455 of a conserved membrane-bound surface protein of *P. malariae* (Figure 3).

**Table 1:** Lists of proteins having the NNLDSKV motif in nature

	Organism name	Protein name	Position
1	SARS-CoV-2	Surface glycoprotein	439–445
2	*Plasmodium malariae*	Conserved surface protein	449–455
3	*Capsella rubella*	GRIP and coiled-coil domain-containing protein 2	1,284–1,290
4	*Pygocentrus nattereri*	Sperm-associated antigen 5 isoform X1	478–484
5	*Lactobacillus gigeriorum*	BspA family leucine-rich repeat surface protein	1,700–1,706
6	*Desulfuromonas* sp. SDB	Hypothetical protein APR63_07190	580–586
7	*Campylobacter concisus*	Retention module-containing protein	1,574–1,580
8	*Nitrospira* sp.	Nonribosomal peptide synthetase	658–664
9	*Smittium simulii*	Hypothetical protein BB561_003344	1,059–1,065
10	*Caldisericales bacterium*	S8 family serine peptidase	510–516
11	*Mizuhopecten yessoensis*	Transient receptor potential cation channel subfamily M member 3-like	1,380–1,386
12	*Bacterium ADurb.Bin132*	Bacillopeptidase F precursor	510–516
13	*Dictyostelium purpureum*	Hypothetical protein DICPUDRAFT_46686	241–247

### 3.3 The predicted T cell epitopes of the conserved surface protein of *P*. *malariae* contain the unique “NNLDSKV” peptide

**Figure 4:**
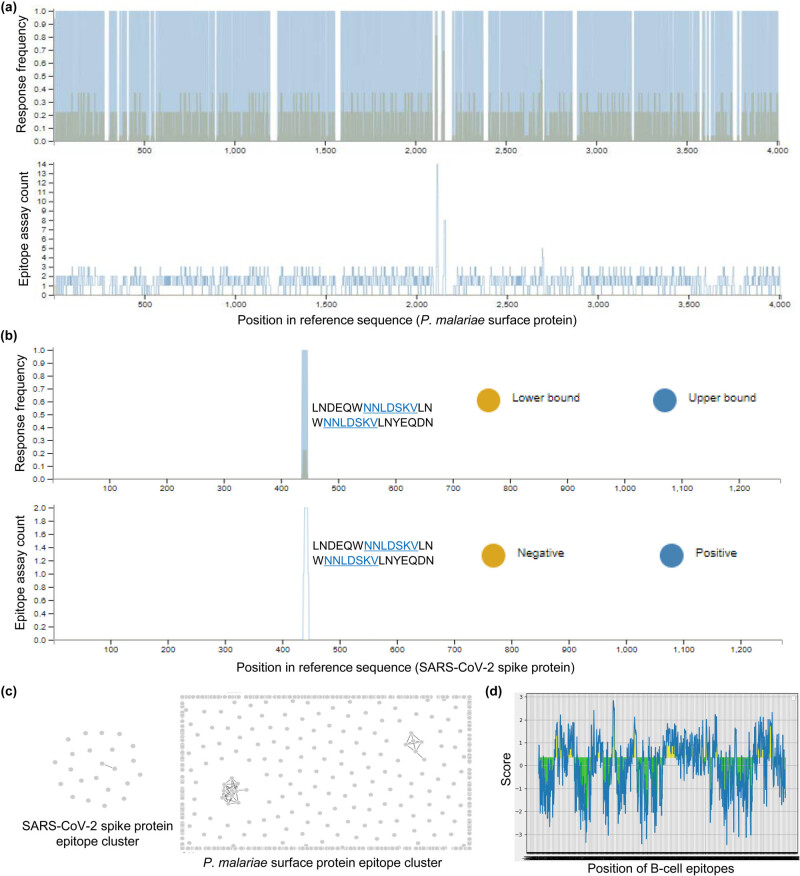
T cell immunodominant regions based on the conserved surface protein of *P. malariae*. (a) Specific T cell epitope mapping response frequency score (RF) for each epitope position from *P. malariae* conserved surface protein. (b) Mapping of T cell epitopes of the conserved surface protein of *P. malariae* against SARS-CoV-2 spike glycoprotein. (c and d) Cluster analysis of epitopes of SARS-CoV-2 spike glycoprotein and surface protein of *P. malariae* for identification sequence homology. (d) Mapping of B cell epitopes from a conserved surface protein of *P. malariae.*

The homology and unique nature of the NNLDSKV motif of SARS-CoV-2 and *P*. *malariae* conserved surface protein let us to investigate the immunogenic nature of NNLDSKV in the conserved surface protein of *P. malariae* T and B cell epitopes. To determine potential T cell epitopes of *P. malariae* conserved surface protein, we used CD4 T cell epitope immunogenicity prediction tool using a combined method embedded in IEDB analysis resource. A total of 801 T cell immunodominant epitopes were identified by defining the threshold at 90%. The response frequency (RF) of T cell epitopes was shown by mapping the epitopes against the parental *P. malariae* conserved surface protein sequence (Figure 4a). Surprisingly, mapping of T cell epitopes of *P. malariae* conserved surface protein against SARS-CoV-2 spike glycoprotein sequence identified two T cell epitopes, ^421^LNDEQWNNLDSKVLN^435^ and ^426^WNNLDSKVLNYEQDN^440^, which showed 97.72 and 98.60% of CD4 T cell immunogenicity and contains peptide core “NNLDSKV” motif (Figure 4b).

We separately performed the cluster analysis of T cell epitopes of SARS-CoV-2 and *P. malariae* conserved surface protein to investigate the sequence similarity within the population. The results showed that only two T cell epitopes of SARS-CoV-2 showed sequence similarity by making one cluster while 17 epitopes of *P*. *malariae* conserved surface protein divided into two different epitope clusters (Figure 4c). To define the B cell epitopes of *P*. *malariae* conserved surface protein, we used the B cell epitope prediction tools provided with IEDB. Using Bepipred method and a threshold of 0.55, the target protein had the highest 122 B cell episodes (Figure 4d). However, the B cell epitopes of *P*. *malariae* conserved surface protein did not show any identity for mapping against the SARS-CoV-2 spike glycoprotein sequence.

### 3.4 The T cell epitopes of SARS-CoV-2 spike glycoprotein showed significant sequence homology with T cell epitopes of the conserved surface protein of *P. malariae*

The SARS-CoV-2 T and B cell epitopes identified by Grifoni et al. [16] were used to investigate the presence of immunodominant epitopes in *P. malariae* conserved surface protein. Before homology analysis, all the T and B cell epitopes of SARS-CoV-2 spike glycoprotein from the previous study were aligned against our prototype SARS-CoV-2 spike glycoprotein sequence, and the aligned sequence showed 100% sequence identity after analysis. The tested (421–435), (426–440), and (841–855) T cell epitope residues of *P. malariae* conserved surface protein showed 44.44, 55.56, and 44.44% homology with (101–118), (304–321), and (440–457) T cell epitope residues of SARS-CoV-2 spike glycoprotein (Table 2). Due to the phylogenetic distance between these two organisms, four or five shared amino acids in a single immunodominant epitope would be considered significant [23]. Surprisingly, two T cell epitopes of SARS-CoV-2 spike glycoprotein (^101^IRGWIFGTTLDSKTQSLL^118^ and ^440^ NLDSKVGGNYNYLYRLFR^457^) shared sequence homology with *P*. *malariae* conserved surface protein T cell epitope by NNLDSKV motif. Conversely, all tested B cell epitopes share no significant homology with SARS-CoV-2 according to the reference sequences of the previous study [16]. In addition to sequence homology, we observed the binding affinity of three different T cell epitopes of *P*. *malariae* conserved surface protein to the 12 most frequent HLA class I alleles in the worldwide population and ranked based on the lowest percentile score, which has the higher binding ability (Table 2).

**Table 2:** Experimental T cell immunodominant epitopes from a conserved surface protein of *P*. *malariae* sharing homology with T cell epitopes of SARS-CoV-2 spike protein

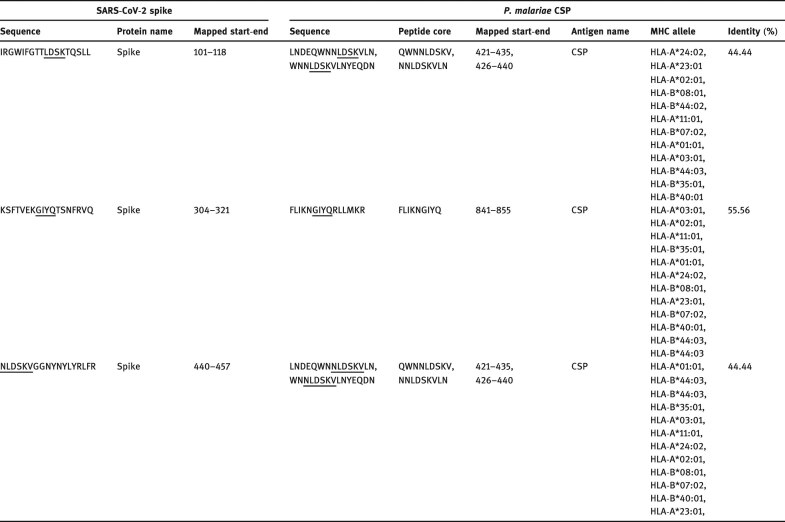

Note: CSP, conserved surface protein; MHC, major histocompatibility complex.

## 4 Discussion

RNA genomes, as in the case of CoVs, are prone to be naturally mutated and thus have a wide range of genetic variation [28,29,30]. Because of the genetic variation between individual strains, it is natural to find that phylogenetic analyses generate different results if different kinds of individual genome sequences were used, explaining current discordant conclusions on the origin of SARS-CoV-2. Since genetic mutation or recombination occurs on an individual basis and these events cannot occur at the same position repeatedly, the prototype sequence of the genome within a species is always the most prevalent base among the members. Also, the degeneracy of codons is present in nucleotide sequences but not in protein aa sequences. Since degenerate codons are not affected by natural selection, there are more genetic variations present in degenerate codons. Considering these natural variabilities of nucleotide sequences in RNA genomes, we believe that our approach of the whole prototype proteome analysis using aa sequence could generate a much more precise result than current approaches in phylogenetic analysis.

The clinical presentation of COVID-19 significantly differs from that of other coronaviruses [31]. The acquisition of the *P. malariae* gene in the spike protein seems to explain why. The malaria-causing *Plasmodium* species infect liver cells and erythrocytes to promote blood clotting and damages in the heart, liver, or kidney. It is fascinating to note that, unlike other coronaviral infections, symptoms similar to the case of the *P. malariae* infection were reported in COVID-19 [33,34,35,36,37]. Red cell distribution width (RDW) of erythrocytes has been reported to be enlarged after the malarial invasion because the growth of the malaria parasite causes the cells to be enlarged [32,33]. It was also observed in COVID-19 that elevated RDW is correlated with the increased mortality risk in COVID-19 [34]. The enlargement of erythrocytes in COVID-19 is not the only factor correlated with malaria infection. Clinical presentations such as blood clotting and damages in the heart, liver, or kidney also were observed in COVID-19 patients [35,36,37]. Considering that, the investigation of different kinds of antimalaria drugs for COVID-19 treatment needs to be pursued.

Several studies have reported the relationship of the ABO blood group system to susceptibility and resistance of excessive *Plasmodium* parasite invasion in severe malaria [38,39]. Individuals with A, B, or AB blood groups are more susceptible to the malarial parasite than O blood group. In agreement with our results, ABO blood group system is also associated with COVID-19, as in malaria. A recent meta-analysis showed that A, B, or AB blood groups are more susceptible to SARS-CoV-2 than O blood group [40,41]. Furthermore, comparative analysis between COVID-19 and malaria showed that malaria-free countries have much higher rates of infectivity and fatality to SARS-CoV-2 compared to malaria-endemic countries [42]. The association between malaria and COVID-19 has suggested that low COVID-19 cases in malaria-endemic countries could be due to the anti-malaria immunity, which provides heterogeneous protection against SARS-CoV-2 [40]. Despite the link between malaria and COVID-19, the reason has not been known. This work suggests that the NNLDSKV motif could be the missing link of malaria and COVID-19. The apparent immunodominant T cell epitope conservation between SARS-CoV-2 spike glycoprotein and conserved surface protein of *P. malariae* may provide immunity against SARS-CoV-2 infection to those previously infected with *Plasmodium*. With that in mind, the possible significance of said motif should be considered during the development of both COVID-19 and malaria vaccines.

According to the current COVID-19 outbreak statistics (https://www.worldometers.info/coronavirus/), a racial background affects the morbidity and mortality of COVID-19. It seems that the morbidity and mortality by COVID-19 are much more severe in the Western world than in Eastern countries. Contrary to the Western, multisystem inflammatory syndrome in children was never reported, and the morbidity and mortality by COVID-19 are much milder in Eastern countries. It would be interesting to investigate the role of the NNLDSKV motif among different racial backgrounds, including ACE-2, since the genetic polymorphism of ACE-2 is known to differ among different ethnic and racial groups [43].
